# Supplementary material for: Scalp recorded theta activity is modulated by reward, direction, and speed during virtual navigation in freely moving humans
Source: Sci Rep. 2022 Feb 7;12:2041. doi: 10.1038/s41598-022-05955-9 (PMC8821620; doi:10.1038/s41598-022-05955-9)
Supplement: Supplementary file 1 — Supplementary Figures. [file 41598_2022_5955_MOESM1_ESM.docx]

**Supplementary Materials:** Scalp recorded theta activity is modulated by reward, direction, and speed during virtual navigation in freely moving humans

**Authors**

Mei-Heng Lin,^1^ Omer Liran,^2^ Neeta Bauer,^1^ Travis E. Baker,^1^*

**Affiliations**

^1^Center for Molecular and Behavioral Neuroscience, Rutgers University, New Jersey

^2^Department of Psychiatry & Behavioral Neurosciences, Cedars-Sinai, California


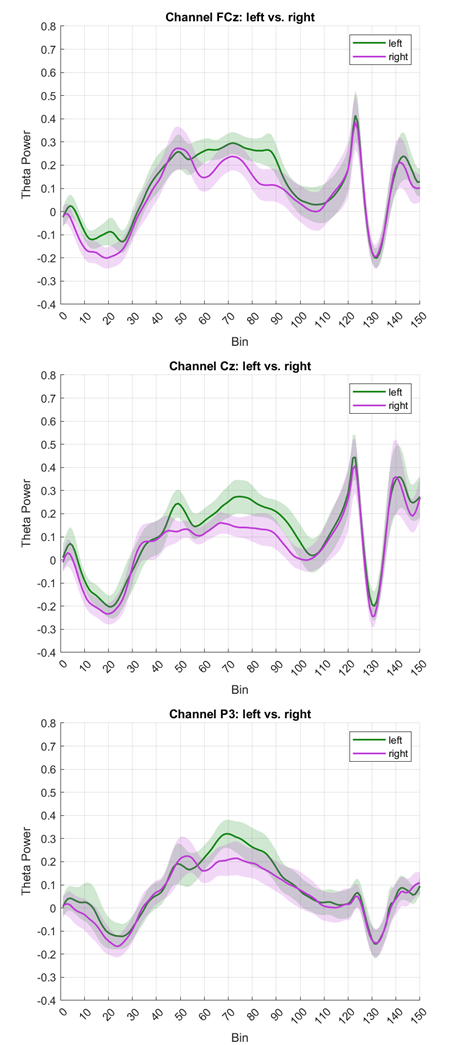


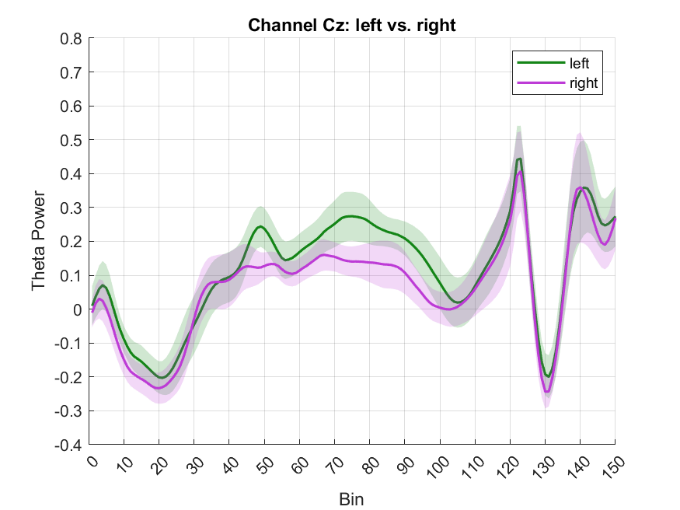

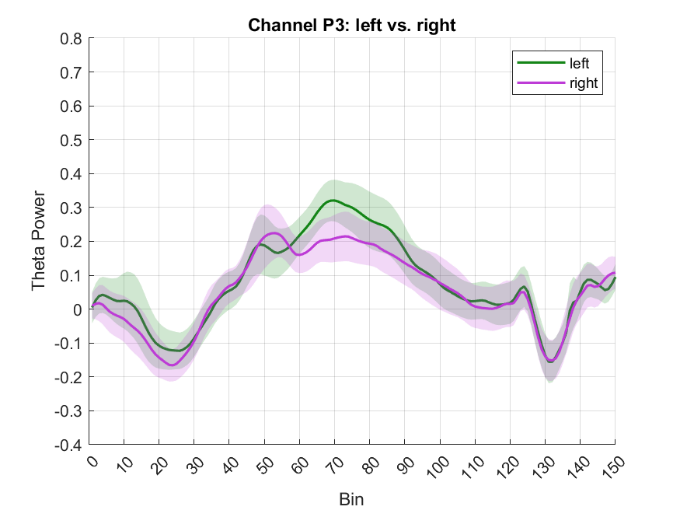


**Figure S1.** **Time-frequency analysis associated with maze trajectories.** For each channel location, FCz (top), Cz (middle), and P3 (bottom), panels depict theta mean time-course for the leftward (green solid lines) and rightward (purple solid lines) conditions. Shaded regions reflect the standard error (SE). The X-axis represents Bin location and Y-axis represents a change in theta power**.** For all conditions, Bin 0 represents the start of the trial, Bin 60 the junction point, and Bin 120 the goal location.


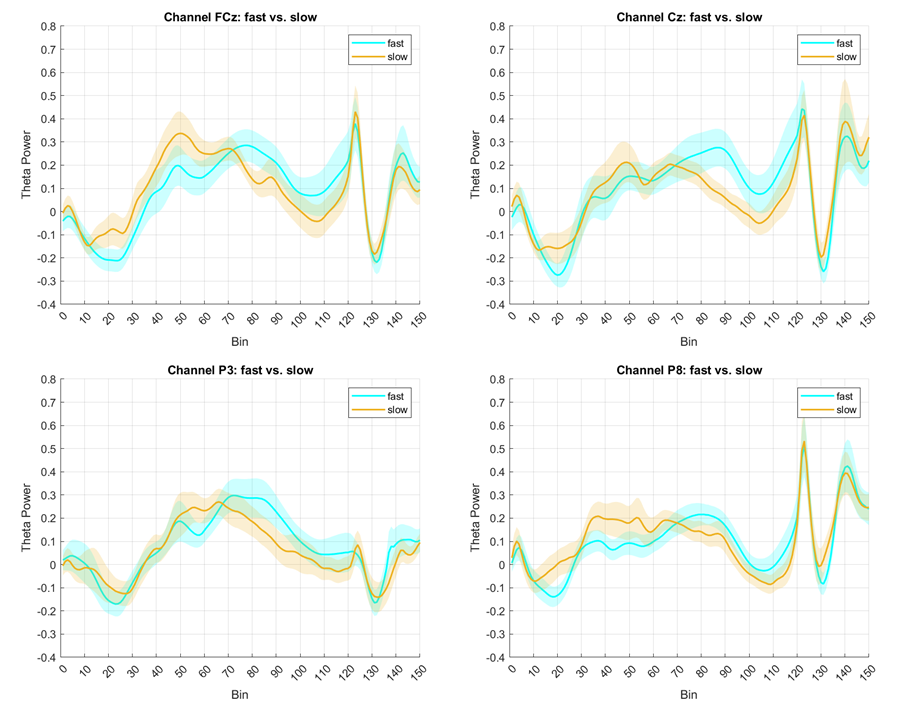


**Figure S2.** **Time-frequency analysis associated with walking speed.** For each channel location, FCz (top-left), Cz (top-right), P3 (bottom-left), and P8 (bottom-right) , panels depict theta mean time-course for the fast (cyan solid lines) and slow (orange solid lines) conditions. Shaded regions reflect the standard error (SE). The X-axis represents Bin location and Y-axis represents a change in theta power**.** For all conditions, Bin 0 represents the start of the trial, Bin 60 the junction point, and Bin 120 the goal location.
